# Changes in MoCA scores with sequential administration of Trikatu Cūrṇa and Brahmi Ghṛta in mild cognitive impairment: an exploratory single-arm clinical study

**DOI:** 10.3389/fmed.2026.1834211

**Published:** 2026-06-19

**Authors:** Saurav Sharma, Devipriya Soman, Sreelekshmi Remadevi, Nagarajan Chockan

**Affiliations:** Department of Kayachikitsa, School of Ayurveda, Amrita Vishwa Vidyapeetham, Amritapuri, India

**Keywords:** Ayurveda, Brahmi Ghṛta, Buddhi bhraṁśa, MCI, MoCA scale, Smṛti bhraṁśa

## Abstract

**Introduction:**

Mild cognitive impairment (MCI) is an intermediate clinical state between normal cognitive aging and dementia. MCI can impair complex daily activities and social functioning. Ayurveda recognizes age-related changes (Jarā) as a natural phenomenon; however, pathological cognitive decline is considered when influenced by doṣa-vaiṣamya. Descriptions of Buddhi-bhraṁśa and Smṛti-bhraṁśa can be correlated with cognitive decline. Current pharmacological and non-pharmacological approaches show variable outcomes, highlighting the need for supportive and integrative strategies.

**Methods:**

The single-arm, open-label clinical study evaluated 24 participants diagnosed with mild neurocognitive disorder according to the Diagnostic and Statistical Manual of Mental Disorders, Fifth Edition (DSM-5) criteria. The patients were administered Trikatu Cūrṇa 3 g twice daily, after food, for 7 days, followed by Brahmi Ghṛta 5 mL twice daily with milk as Anupāna, after food, for 90 days. Out of the 27 enrolled participants, 24 completed the study and 3 dropped out. Cognitive assessment was carried out using the Montreal Cognitive Assessment (MoCA) Scale. This study is registered in the Clinical Trial Registry of India under CTRI/2024/07/070223 before enrolment of the first participant.

**Results:**

Post-intervention assessment demonstrated a statistically significant improvement in overall cognitive scores compared to baseline (*p* < 0.001), with a large effect size and no adverse events. However, these findings are strictly limited by the open-label, single-arm design, lack of lifestyle standardization, and unrecorded concomitant medications.

**Discussion:**

Trikatu Cūrṇa, through its Dīpana–Pācana action, may support Agni and help in Āma reduction, thereby improving metabolic efficiency and drug absorption. Brahmi (*Bacopa monnieri*) formulation, Brahmi Ghṛta, is traditionally described as a Medhya Rasāyana, and based on experimental and pharmacological evidence, it may support cognitive function through mechanisms such as modulation of cholinergic pathways, enhancement of synaptic plasticity, and neuroprotective effects in hippocampal circuits relevant to MCI.

**Clinical trial registration:**

https://ctri.nic.in/Clinicaltrials/pmaindet2.php?EncHid=MTEwODI2&Enc=&userName=.

## 1 Introduction

Mild cognitive impairment (MCI) is a neurocognitive disorder that represents an intermediate stage between normal age-related memory changes and dementia. Individuals with MCI exhibit more memory or thinking difficulties than expected for their age and educational background (1). As the aging global population is accelerating rapidly (2), the burden of MCI is increasing worldwide. Epidemiological data indicate that 12–18% of individuals aged above 60 years are affected globally, with even higher prevalence reported in low- and middle-income countries, including India, where estimates range from 15 to 26% depending on demographic and regional factors (3–8). Several studies indicate that patients with MCI have a higher risk of developing Alzheimer’s, with annual conversion rates estimated at 3–10% in community settings and 10–15% in specialized clinics (9).

Although individuals with MCI maintain independence, subtle impairment in memory, attention, and executive functions can interfere with complex daily activities such as financial management, medication routines, and social participation, along with having a psychosocial impact (10, 11). Despite extensive research, current therapeutic options for MCI remain limited. Pharmacological agents such as cholinesterase inhibitors and memantine have been evaluated, but the evidence regarding their efficacy in delaying progression to dementia still remains inconsistent (3, 12). A Cochrane systematic review including over 5,000 individuals with MCI concluded that there is very little evidence that cholinesterase inhibitors reduce progression to dementia or improve cognitive test scores. Although a pooled analysis suggested a possible short-term reduction in conversion risk, this finding was based on limited data, lacked corresponding cognitive improvement, and was offset by significantly higher rates of adverse events, raising concerns about long-term tolerability and adherence (12).

Similarly, non-pharmacological interventions, including cognitive training, physical activity, and dietary modifications, have shown variable benefits, with outcomes influenced by heterogeneity in study design and intervention protocols (13, 14). Thus, the current therapeutic approaches are not reliable enough, as they fail to produce clinically significant improvement either in cognition or independence for most patients (15).

Ayurveda describes cognitive function through the integrated roles of Buddhi (intellect and decision-making) and Smṛti (memory) and regards cognitive decline as a clinically and prognostically significant process rather than a benign consequence of aging. Classical descriptions state that impairment of higher cognitive functions is influenced by aging, lifestyle factors, and metabolic imbalance. Ayurvedic texts further recognize a gradual age-related decline in cognitive capacities such as grahaṇa (perception), dhāraṇa (retention), and smaraṇa (recall), which parallels the concepts of age-related cognitive decline. Importantly, Ayurveda distinguishes between physiological cognitive aging and pathological decline, emphasizing that when age-related changes are compounded by doṣic imbalance and adverse lifestyle factors, cognitive deterioration progresses toward a disease state.

Ayurveda describes Medhya Rasāyana therapy as a strategy for supporting cognitive function and mitigating age-related cognitive decline. Classical principles state that Rasāyana interventions should be preceded by Śodhana; however, in elderly populations, Dīpana and Pācana are used to restore metabolic efficiency and enhance therapeutic responsiveness. Trikatu Cūrṇa, a polyherbal formulation traditionally indicated for improving digestion, reducing metabolic byproducts (Āma), and balancing Kapha and Vata, is employed to optimize systemic metabolism before Rasāyana administration. Brahmi Ghṛta is a Medhya Rasāyana formulation traditionally indicated for disorders involving memory, intellect, and higher cognitive functions, including Buddhi Māndya and Smṛti Kṣaya.

The present study was therefore undertaken to evaluate the effect of Dīpana–Pācana with Trikatu Cūrṇa, followed by the administration of Brahmi Ghṛta on cognitive function in individuals with MCI, assessed using the Montreal Cognitive Assessment (MoCA). This exploratory pre-post clinical study aims to generate preliminary clinical evidence for an integrative therapeutic approach to early cognitive decline.

## 2 Methods

### 2.1 Study design and ethical considerations

This is a single-arm, open-label, pre- and post-test study. Participants were selected from the outpatient department (OPD) and inpatient department (IPD) of Amrita School of Ayurveda, Kollam, Kerala, and the study was conducted between July 2024 and October 2025. A tabular presentation of the methodology is provided (Table 1). The Clinical Research Act (enacted April 14, 2017) and the amended October 2013 Declaration of Helsinki have been followed in this investigation. Furthermore, all relevant regional, national, and international laws have been followed. This study is registered in the Clinical Trial Registry of India under CTRI/2024/07/070223 before the enrolment of the first participant. The MoCA scale was administered by a trained assessor who completed MoCA certification training through the official MoCA platform (mocacognition.com) (MoCA Certified Rater ID: INSHASA710723384-01). All assessments were conducted by the same qualified individual; therefore, inter-rater reliability testing was not required, ensuring perfect consistency in scoring application across all study participants.

**Table 1:** Methodology.

Steps	Phase	Detail
Step 1	Preparatory phase	Study was planned, CRF and informed consent form prepared, IEC clearance obtained, CTRI registration done, study drug procured from a GMP-certified pharmacy.
Step 2	Screening and recruitment	Patients with MCI screened as per inclusion/exclusion criteria Eligible participants selected
Step 3	Baseline assessment (Day 0)	Informed consent obtained Detailed case history and clinical examination Baseline MoCA scoring Enrolment of participants
Step 4	Intervention: Phase I (Day 1–Day 7)	Trikatu Cūrṇa administered for 7 days (Dīpana and Pācana)
	Intervention: Phase II (Day 8 onwards)	Brahmi Ghṛta started after 7 days of Phase I
Step 5	Revisits (every 15 days)	Participants submitted empty bottles New medication supplied Compliance monitored Monitored for adverse events
Step 6	Final assessment (on 98th day)	Reassessed with MoCA scoring after 97 days of intervention (Trikatu Cūrṇa, 7 days, and Brahmi Ghṛta, 90 days) Outcomes documented in CRF

CRF, case report form; CTRI, Clinical Trial Registry of India; GMP, Good Manufacturing Practice; IEC, institutional ethics committee; MCI, mild cognitive impairment; MoCA, Montreal Cognitive Assessment.

### 2.2 Participant selection

The participants were selected based on the Diagnostic and Statistical Manual of Mental Disorders, Fifth Edition (DSM-5) diagnostic criteria (16). The senior clinical researcher in our team diagnosed the condition. Informant history was considered to know the accurate status of functional and social independence and financial dealings of the participant. Moreover, Mini-Mental State Examination was included as a baseline filter to assess the status of cognition and to exclude higher cognitive decline and dementia.

#### 2.2.1 Inclusion criteria

- Participants diagnosed with MCI according to DSM-5 criteria between 60 and 80 years of age of either gender who were willing to take part in the study.
- MoCA scale assessment scores ranging from 16 to 26 points.

#### 2.2.2 Exclusion criteria

The patients with the following conditions were excluded:

- those with pre-diagnosed neurological (seizures, vascular accidents, Parkinson’s disease) and psychiatric diseases (schizophrenia, mania, depression, delirium),
- those demonstrating any loss of functional autonomy or global cognitive degradation indicative of dementia,
- those with pre-diagnosed chronic infection,
- those with pre-diagnosed thyroid disorders,
- those with electrolyte imbalance,
- those who were on cholinesterase inhibitors or glutamatergic drugs,
- those on medication with fasting plasma glucose levels above 126 mg/dL and uncontrolled postprandial levels above 160 mg/dL,
- those on medication with blood pressure above 150/90 mmHg, or
- those on medication with total cholesterol levels above 190 mg/dL.

### 2.3 Enrolment and intervention

Participants diagnosed with MCI were screened and enrolled from the OPD and IPD after fulfilling the predefined inclusion and exclusion criteria. Written informed consent was obtained from all participants before enrolment. The total study duration for each participant was 97 days, and baseline clinical assessment and cognitive evaluation were performed before initiation of the intervention.

Following enrolment, the intervention was administered in two sequential phases in accordance with classical Ayurvedic principles. In the initial phase, Trikatu Cūrṇa was prescribed at a dose of 3 g twice daily (BD) with lukewarm water for a duration of 7 days, with the objective of achieving Dīpana and Pācana. After completion of the initial phase, participants were administered Brahmi Ghṛta at a dose of 5 mL twice daily (BD) with milk for a period of 90 days. The Ghṛta was given orally, following standard clinical practice. Compliance with the intervention was assessed during scheduled follow-up visits conducted at 15-day intervals, during which returned containers were reviewed to verify medication intake. Participants were also monitored throughout the study period for the occurrence of any adverse events.

### 2.4 Outcome measures

Cognitive outcome was evaluated by the change in total scores (pre- and post-intervention) on the MoCA scale, indicating changes in overall cognitive performance. MoCA was selected in the present exploratory study because of its feasibility, clinical practicality, sensitivity to mild cognitive changes, and suitability within the available study setting.

### 2.5 Operational protocol and blinding

Absolute blinding of investigators and participants was unfeasible as the study was a single-arm, open-label trial. The clinical researcher administering the post-intervention MoCA on Day 98 was aware of the treatment timing and intervention status.

### 2.6 Sample size

- To determine the required sample size for this exploratory, single-arm, pre-post study, a power analysis was conducted based on the primary outcome measure: the change in the total MoCA score from baseline to post-intervention.
- Since the study used a paired, within-subject design, the standard sample size formula for evaluating paired differences was applied:

$$n=\frac{{\left[Z_{1-\alpha /2}+Z_{1-\beta }\right]}^{2}}{\Delta^{2}}\sigma^{2},$$

where

- *Δ* = $\left\|\mu_{1}-\mu_{2}\right\|$
- *α* = Level of significance
- 1 - *β* = Power of the test
-
$\mu_{1}$ = Pre-test mean
-
$\mu_{2}$ = Post-test mean
-
σ = Standard deviation (SD) of the within-subject pre-post differences

To obtain an estimator for sample size n, we assume that the SD of the differences to be (σ) = 3, following historical variance thresholds established during exploratory 12-week tracked protocols evaluating Brahmi Ghṛta tracking indices in early cognitive decline (17), and the expected mean change in the total MoCA score from baseline to post-intervention was conservatively set as (Δ) = 2. Then, for a fixed level of significance α = 0.05 and power 1 - β = 0.80, the estimated sample size was n = 18. If the dropout rate (d) was 25%, N = $\frac{n}{1-d}$ . That is, N = 24.

### 2.7 Trial drugs

#### 2.7.1 Trikatu Cūrṇa

Trikatu Cūrṇa was procured from the Good Manufacturing Practice (GMP)-certified factory of Amrita Life, Kollam, Kerala.

#### 2.7.2 Brahmi Ghṛta

Brahmi Ghṛta was procured from the GMP-certified factory, batch no. BRG11794, of Amrita Life, Kollam, Kerala.

### 2.8 Statistical analysis

Data were entered and analyzed during the initial phase using Microsoft Excel. Further statistical analyses, including testing for normality and hypothesis testing using Wilcoxon signed-rank analysis and Rosenthal’s *r* effect size for the Wilcoxon signed-rank test, were performed in RStudio version 2026.04.0 + 526. The *readxl*, *coin*, and *rstatix* packages were used in the aforementioned statistical software for the analysis.

Normality was assessed using the Shapiro–Wilk test due to the small sample size (n = 24). The change in total MoCA score, designated as the primary outcome measure, deviated from normality and was therefore further assessed with the Wilcoxon signed-rank test. The precision of the location shift was quantified by reporting the 95% confidence interval for the pseudo-median difference based on the non-parametric Hodges–Lehmann estimator. The magnitude of the treatment effect was calculated using Rosenthal’s *r* effect size formula (r = Z/√N). Effect size thresholds were interpreted according to Cohen’s criteria. All statistical tests were two-tailed, and a *p*-value of < 0.05 was considered statistically significant.

### 2.9 Safety and adverse-event monitoring

Safety and tolerability profiles were tracked using active clinical surveillance rather than passive participant reporting. Adverse events (AEs) were actively solicited during scheduled physical evaluations, at the conclusion of the Trikatu phase (Day 7), during each visit scheduled at an interval of 15 days, and at the terminal assessment (Day 98). At each visit, clinicians utilized a structured, non-suggestive interview protocol to screen for gastrointestinal symptoms common to lipid-based Ayurvedic formulations—including nausea, dyspepsia, hyperacidity, and altered bowel habits—alongside routine vital sign tracking. No serious adverse events or significant metabolic intolerances were reported or clinically observed over the 97-day protocol.

## 3 Results

**Figure 1:**
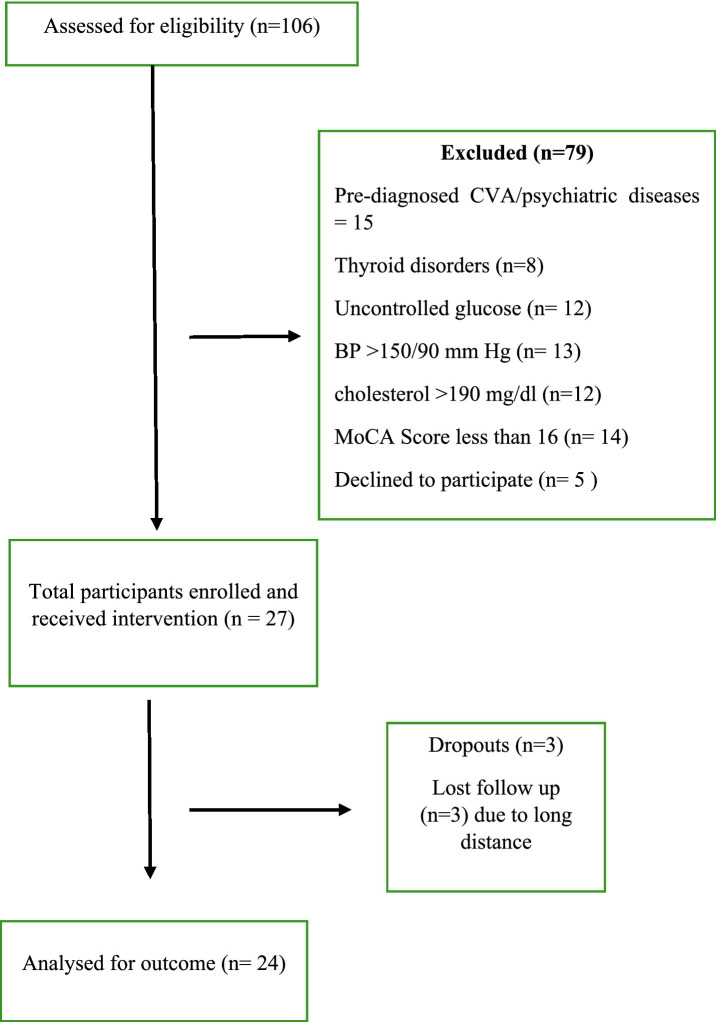
Participant flow chart. BP, blood pressure; CVA, cerebrovascular accident; MoCA, Montreal Cognitive Assessment.

A total of 106 individuals were assessed for eligibility. Of these, 79 were excluded based on predefined criteria. During the study period, three participants were lost to follow-up due to logistic reasons. Consequently, data from 24 participants were available and included in the final analysis of outcomes (Figure 1).

### 3.1 Baseline data

The study population predominantly comprised individuals between 60 and 75 years of age, with the largest proportion (37.5%) belonging to the 70–75-year age group. Female individuals constituted approximately two-thirds of the participants. A majority of the individuals belonged to the middle socioeconomic category (54.1%), followed by the lower socioeconomic group, whereas representation from the upper socioeconomic class was minimal. Educational attainment was generally low to moderate, with half of the participants having completed 5–9 years of formal education and fewer individuals reporting higher levels of education. Slightly more than half of the participants followed a mixed diet, while the remainder consumed a vegetarian diet. Irregular appetite and bowel habits were commonly reported, reflecting underlying digestive irregularities, whereas sleep patterns were largely preserved, with nearly two-thirds of participants reporting normal sleep (Table 2).

**Table 2:** Baseline data.

Characteristics	*N* (%)
Age (years)	
60–65	7 (29.1%)
65–70	5 (20.8%)
70–75	9 (37.5%)
75–80	3 (12.5%)
Sex	
Female	16 (66.6%)
Male	8 (33.3%)
Socioeconomic status	
Lower	9 (37.5%)
Middle	13 (54.1%)
Upper	2 (8.3%)
Years of education	
5–9	12 (50.0%)
9–13	9 (37.5%)
13–17	2 (8.3%)
17–21	1 (4.1%)
Diet	
Mixed	13 (54.1%)
Veg	11 (45.8%)
Appetite	
Irregular	14 (58.3%)
Poor	10 (41.6%)
Bowel	
Irregular	15 (62.5%)
Regular	9 (37.5%)
Sleep	
Disturbed	9 (37.5%)
Normal	15 (62.5%)

### 3.2 Assessment of normality

The difference between the pre- and post- total MoCA scores was tested for normality using the Shapiro–Wilk test. The score (W = 0.914, *p* = 0.044) deviated from normality; hence, the Wilcoxon signed-rank test was used for further analysis.

### 3.3 Statistical analysis

Baseline descriptive statistics and pre- and post-intervention changes in mean with SD and median with interquartile range cognitive scores are summarized in Table 3.

**Table 3:** Descriptive analysis.

Parameters	Time point	*N*	Mean (±SD)	Median	IQR	Range (Min–Max)
MoCA score	Before	24	18.88 (±2.58)	18.50	4.50 (16.25–20.75)	7 (16–23)
	After	24	24.50 (±1.82)	25.00	3.00 (23.00–26.00)	7 (20–27)
Visuospatial	Before	24	0.54 (±1.06)	0.00	1.00 (0.00–1.00)	4 (0–4)
	After	24	0.54 (±1.06)	0.00	1.00 (0.00–1.00)	4 (0–4)
Naming	Before	24	2.25 (±0.53)	2.00	1.00 (2.00–3.00)	2 (1–3)
	After	24	2.83 (±0.38)	3.00	0.00 (3.00–3.00)	1 (2–3)
Digit span	Before	24	1.08 (±0.65)	1.00	0.75 (1.00–1.75)	2 (0–2)
	After	24	1.88 (±0.34)	2.00	0.00 (2.00–2.00)	1 (1–2)
Vigilance	Before	24	0.58 (±0.50)	1.00	1.00 (0.00–1.00)	1 (0–1)
	After	24	1.00 (±0.00)	1.00	0.00 (1.00–1.00)	0 (1–1)
Serial 7 subtraction	Before	24	1.63 (±1.35)	2.00	3.00 (0.00–3.00)	3 (0–3)
	After	24	2.33 (±0.82)	3.00	1.00 (2.00–3.00)	2 (1–3)
Sentence repetition	Before	24	1.71 (±0.55)	2.00	0.75 (1.25–2.00)	2 (0–2)
	After	24	1.83 (±0.48)	2.00	0.00 (2.00–2.00)	2 (0–2)
Verbal fluency	Before	24	0.67 (±0.56)	1.00	1.00 (0.00–1.00)	2 (0–2)
	After	24	1.00 (±0.00)	1.00	0.00 (1.00–1.00)	0 (1–1)
Abstraction	Before	24	1.38 (±0.65)	1.00	0.75 (1.00–2.00)	2 (0–2)
	After	24	1.75 (±0.44)	2.00	1.00 (1.25–2.00)	1 (1–2)
Delayed recall	Before	24	2.71 (±1.33)	3.00	2.00 (2.00–4.00)	5 (0–5)
	After	24	4.33 (±0.87)	5.00	1.00 (4.00–5.00)	3 (2–5)
Orientation	Before	24	5.71 (±0.62)	6.00	0.00 (6.00–6.00)	2 (4–6)
	After	24	5.92 (±0.28)	6.00	0.00 (6.00–6.00)	1 (5–6)

IQR, interquartile range; MoCA, Montreal Cognitive Assessment; N, number of participants; SD, standard deviation.

A total of 24 participants completed the study and were included in the final analysis. Cognitive performance was assessed before and after the intervention using the MoCA. A standard 1-point correction was applied to the total MoCA score for all participants with 12 years or fewer of formal education, provided that the total score did not exceed 30. This correction was applied at the final stage of total score calculation.

At baseline, participants demonstrated impaired cognitive function, with a mean MoCA score of 18.88 ± 2.58. Following the intervention, the mean MoCA score increased to 24.50 ± 1.82, representing an overall mean improvement of +5.63 points. The Wilcoxon signed-rank test revealed a statistically significant improvement in total MoCA scores post-intervention with Z = 4.305 and *p* < 0.001. The analysis confirmed a significant positive location shift, yielding a Hodges–Lehmann pseudo-median paired difference of 5.63 points with a corresponding 95% confidence interval of 3.60 to 7.65. The Rosenthal’s *r* effect size for the Wilcoxon signed-rank test indicated a large effect size of 0.879.

A domain-wise analysis demonstrated numerical upward trends across several MoCA cognitive domains following the intervention. As inferential hypothesis testing was restricted to the primary outcome measure (total MoCA score), these subdomain observations should be interpreted as exploratory descriptive patterns rather than statistically verified domain-specific effects. The mean score of naming (Table 3) showed an increase, indicating improved lexical retrieval. Attention-related domains demonstrated consistent gains following the intervention. In the digit span task (Table 3), mean performance (mean score) improved, suggesting improvement in working memory and immediate attention. Serial 7 subtraction (Table 3) mean scores also increased, reflecting improved concentration and mental calculation ability. Vigilance (Table 3), assessed through the alphabet tapping task, showed improvement in mean scores.

Language functions exhibited modest changes. The mean scores of verbal fluency (Table 3), improved, whereas sentence repetition (Table 3) showed minimal variation. While the mean scores of abstractions (Table 3) improved, with participants demonstrating better conceptual reasoning after the intervention, the mean scores of delayed verbal recall (Table 3) also showed improvement, with gains in memory retention. Orientation scores remained mostly stable, with high baseline performance. Visuospatial performance remained unchanged throughout the study period.

### 3.4 Descriptive profile of cognitive subdomains

To provide a granular overview of the cohort’s performance across the multi-phase protocol, baseline and post-intervention descriptive statistics for the individual MoCA subdomains are detailed in Table 3. In alignment with the global screening design of the MoCA and to prevent the inflation of Type I errors through multiple comparisons, formal inferential hypothesis testing was restricted exclusively to the primary outcome measure, the total MoCA score.

Qualitatively, minor numerical increases were noted across several subscale scores, including attention, memory, and language. Conversely, executive functions remained unchanged. These descriptive trends are further interpreted strictly as exploratory baseline features of our sample and are not indicative of statistically verified, selective domain-specific therapeutic effects.

## 4 Discussion

The present study evaluated the effect of a sequential Ayurvedic intervention consisting of Dīpana–Pācana with Trikatu Cūrṇa, followed by the administration of Brahmi Ghṛta on cognitive function in individuals with MCI. The findings demonstrated a significant improvement in overall cognitive performance, as reflected by the increase in total MoCA scores following the intervention.

MCI can be understood as a Vāta-predominant, Tridoṣaja functional disorder. Ayurveda describes aging as being associated with progressive reduction in Grahaṇa, Dhāraṇa, and Smaraṇa śakti, which becomes pathological when vitiated by doṣic imbalance (Caraka Vimānasthāna 8/122) (18). The disturbed Prāṇa Vāyu disrupts its role in regulating mental functions, sensory integration, and higher cognitive processing, while functional impairment of Sādhaka Pitta, located in the Hṛdaya, results in diminished intellectual clarity, judgment, and memory (Astang Hṛdaya Sūtrasthāna 12) (19). Additionally, Tarpaka Kapha, responsible for nourishing and stabilizing neural structures, becomes functionally compromised, contributing to cognitive dullness and reduced processing speed. With advancing age, Agni declines, leading to Agnimāndya, which influences the dhātu sāratā, contributing to cognitive decline. The pathological process progresses through Rasavaha Srotas dysfunction, leading to inadequate nourishment of neural tissues, and Manovaha Srotas is also involved in the process, impairing the coordinated interaction between mind, senses, and intellect required for normal cognition (Caraka Sūtrasthāna 11/20) (18). The resultant Doṣa–Duṣya interaction at the level of Hṛdaya and Manovaha Srotas manifests as an impairment of Buddhi and Smṛti rather than their complete loss, corresponding to classical descriptions of Buddhi bhraṁśa and Smṛti bhraṁśa (Caraka Sūtrasthāna 1/99; Śārīrasthāna 1/101) (18), which explains the gradual, selective cognitive decline with preserved functional independence characteristic of MCI and provides a rational basis for therapeutic strategies emphasizing metabolic correction (Dīpana–Pācana) before cognitive-enhancing interventions (Medhya Rasāyana).

In the present study, administration of Brahmi Ghṛta resulted in statistically significant and clinically meaningful improvement in total MoCA score, supported by both inferential statistics and large effect sizes. These findings are consistent with prior randomized controlled trials and meta-analytic evidence indicating that *Bacopa monnieri* exerts its most reliable cognitive effects on attentional processing and verbal learning–memory functions, particularly delayed recall and retention of newly acquired information (20–22). Meta-analyses have demonstrated reproducible improvements in attention speed and memory consolidation, while individual trials report benefits in verbal learning and delayed recall measures such as the auditory verbal learning test (23, 24). Morgan and Stevens reported significant improvements in verbal learning and delayed recall following *Bacopa monnieri* administration, while performance on the Rey–Osterrieth complex figure test did not differ from placebo (24). Systematic reviews further support this domain-specific pattern, demonstrating consistent benefits in attention and verbal memory, with variable and less reliable effects on visuospatial or visual working memory domains (21–23, 25).

In the present study, Trikatu Cūrṇa was administered before Brahmi Ghṛta to optimize metabolic readiness for Rasāyana therapy. Ayurveda states that Rasāyana should be administered only in a nirāma state, as Āma and impaired Agni hinder digestion, absorption, and tissue-level efficacy (“rasāyanānām prayogo nirāmaḥ;” Cikitsāsthāna 1) (18). Although Śodhana is traditionally recommended, it was avoided due to the advanced age of participants (60–80 years), considering age-related decline in dhātu, Agni, and śarīrabala (Caraka Vimānasthāna 8) (18) and increased physiological vulnerability in geriatric populations (26). These classical considerations align with modern evidence of reduced digestive capacity and metabolic efficiency in old age (27–29). Trikatu Cūrṇa bioactive constituents include piperine, gingerols, and shogaols and have demonstrated effects on gastric secretion, gut motility, enzymatic activity, splanchnic circulation, and nutrient bioavailability, supporting improved digestive efficiency and systemic absorption (30–35). This serves as metabolic priming, which was essential to ensure effective digestion, absorption, and therapeutic delivery of Brahmi Ghṛta. The primary alkaloid, piperine, acts as an epithelial bio-enhancer that modulates tight junction integrity, thereby augmenting the absorption and systemic bioavailability of Brahmi Ghṛta (36). The active components of Trikatu exert prebiotic-like effects that favorably alter gut microbiota composition; this shifts metabolic output toward short-chain fatty acids (SCFAs), which cross the blood–brain barrier to attenuate microglial activation and support hippocampal health (37, 38). The compounds act as agonists for receptors such as enteric transient receptor potential vanilloid-1 (TRPV1), triggering vagal afferent activation that downregulates peripheral pro-inflammatory cytokines and buffers the central nervous system against neuroinflammatory cognitive decline (39). Consequently, Trikatu likely serves as an indirect metabolic and anti-inflammatory optimizer of cognitive function via this bidirectional axis.

Brahmi Ghṛta is a classical Medhya Rasāyana described in Aṣṭāṅga Hṛdaya (Uttara Sthāna, Unmāda Cikitsā) (19), traditionally indicated for disorders of Manas and to support Buddhi and Manas through Tridoṣa-śamana. In addition, Ghṛta is characterized as an ājāsrika Rasāyana and influences Dhi, Smṛti, and Medhā (Aṣṭāṅga Hṛdaya, Uttara Sthāna 16/8) (19). *Bacopa monnieri* exerts cognitive effects via bacosides A and B, which modulate cholinergic transmission, enhance synaptic plasticity, and support dendritic integrity in hippocampal circuits relevant to MCI (40–46). Ghṛta serves as a lipid delivery system enhancing solubility, absorption, and potential central nervous system access of lipophilic phytoconstituents, including bacosides, with emerging evidence suggesting facilitation of blood–brain barrier passage and superior neurocognitive outcomes compared to non-lipid formulations (47–54).

The post-intervention evaluation revealed a large effect size for the total MoCA score (0.879). While this finding confirms a highly uniform upward trajectory in cognitive performance metrics across our participants, these findings should be interpreted with academic caution. In a single-arm, open-label design lacking a parallel control group, calculated effect sizes can be heavily inflated by confounding variables. These metrics likely capture a combined effect stemming from the practice and learning curve of repeated MoCA exposure, the Hawthorne effect of regular clinical monitoring, and the cumulative therapeutic impact of the initial Trikatu baseline priming phase.

### 4.1 Limitations of the study

While the findings of this study offer valuable preliminary observations regarding cognitive changes following Brahmi Ghṛta administration, several methodological limitations should be considered when interpreting the data.

#### 4.1.1 Uncontrolled design, the single-center open-label setting

Foremost, the single-arm, open-label nature of this trial poses substantial threats to internal validity. In the absence of a randomized parallel control or placebo group, it is impossible to determine a definitive causal relationship between the intervention and the observed changes. The improvements may be heavily influenced by the “practice effect” of repeated MoCA administration, natural history, regression to the mean, or the “Hawthorne effect” driven by regular clinical attention. Furthermore, being a single-center study with a small sample size, the generalizability of these findings to a broader, demographically diverse population remains limited.

#### 4.1.2 Inability to disentangle the effects of Trikatu from Brahmi Ghṛta

This study utilized a multi-phase sequential protocol; hence, we cannot disentangle the independent therapeutic contributions of the initial Trikatu priming phase from those of the Brahmi Ghṛta phase. The post-intervention metrics reflect the cumulative trajectory of both phases, and it remains unclear if the cognitive shifts were initiated or amplified primarily by the preparatory metabolic adjustment or the core lipid formulation.

#### 4.1.3 Absence of longer follow-up

The final cognitive evaluations were completed on Day 98, immediately following the termination of the treatment window. The lack of a long-term wash-out or follow-up period prevents us from determining whether the observed score variations are sustainable over time or if they decay once the active administration of Brahmi Ghṛta ceases.

#### 4.1.4 Distinction between statistical significance and clinically meaningful change

Although the primary analysis demonstrated highly robust statistical significance (*p* < 0.001), (Z = 4.305) and Rosenthal’s *r* effect size for the Wilcoxon signed-rank test (r = 0.879), statistical power should not be conflated with uniform, clinically meaningful change. The MoCA is optimized as a global screening instrument rather than a granular neuropsychological battery. Subtle numerical score increases on a screening tool, while statistically verified, do not necessarily guarantee profound improvements in a participant’s daily qualitative functioning or underlying neuropathological disease course.

#### 4.1.5 Limits to generalizability

The generalizability of our findings is significantly constrained by the extensive and stringent exclusion criteria required by our study protocol. While these strict boundaries were essential to eliminate major confounding variables and preserve a homogeneous mild neurocognitive disorder cohort, they created an idealized sample that does not reflect the complex, multimorbid profiles typically encountered in routine clinical practice. Consequently, the observed cognitive trends cannot be widely generalized to the broader population of individuals with mild neurocognitive decline, who frequently present with overlapping chronic medical conditions and polypharmacy.

#### 4.1.6 Analysis population and blinding

Finally, the statistical findings of this study are limited by a completers-only analysis architecture. The clinical assessor administering the post-treatment MoCA was not blinded to the study timing or the participant’s treatment status, which is an operational limitation that could introduce observer expectancy bias.

#### 4.1.7 Unmonitored confounders and medical logs

The confounding factors, including lifestyle and diet, were not standardized in the trial. This introduces a potential unmeasured confounding effect. In addition, the concomitant medical logs were not monitored. Thus, the potential influence of concurrent drug regimens or undocumented drug–herb interactions on the observed cognitive outcomes cannot be entirely ruled out.

Consequently, this trial should be viewed strictly as an exploratory, hypothesis-generating study. These preliminary trends require validation through future large-scale, multi-center, double-blind, randomized placebo-controlled trials featuring long-term longitudinal tracking.

## 5 Conclusion

MoCA assessment showed post-intervention improvement. MoCA subdomains for attention, memory, and language showed numerical upward trends, while executive function remained stable. Since statistical testing was limited to the total MoCA score, these subdomain trends are strictly exploratory. The intervention was well tolerated, with no reported adverse effects, supporting its safety in the elderly population. The study supports the classical principle that Rasāyana therapy should be preceded by Dīpana and Pācana. Administration of Trikatu Cūrṇa before Brahmi Ghṛta may have improved Agni and reduced Āma, thereby facilitating better absorption and therapeutic action of the Rasāyana. Brahmi Ghṛta, being a Medhya Rasāyana, appears to contribute to the enhancement of Medhā, Smṛti, and Buddhi, which are central to cognitive functioning.

Although the study was limited by its single-arm pre–post design, small sample size, and lack of a control group, the findings provide preliminary clinical evidence supporting the usefulness of this classical Ayurvedic treatment protocol in MCI. The improvement in MoCA scores may be attributed to practice effects from repeated testing, the confounding influence of the initial Trikatu phase, or the non-specific effects of clinical attention. The results of this study lay a foundation for future randomized controlled trials with larger sample sizes and longer follow-up, integrated with cognitive behavioral therapies, which may further establish the role of Trikatu Cūrṇa and Brahmi Ghṛta in the management of MCI.

## Data Availability

The original contributions presented in the study are included in the article/supplementary material, further inquiries can be directed to the corresponding author.
